# The reality of using primaquine

**DOI:** 10.1186/1475-2875-9-376

**Published:** 2010-12-27

**Authors:** Kathy L Burgoine, Germana Bancone, François Nosten

**Affiliations:** 1 Shoklo Malaria Research Unit, Mae Sot, Tak, Thailand, 63110; 2 University College, The University of Oxford, Oxford, UK; 3 Mahidol-Oxford Tropical Medicine Research Unit (MORU), Faculty of Tropical Medicine, Mahidol University, Bangkok, Thailand; 4 Centre for Clinical Vaccinology and Tropical Medicine, Headington, Oxford, UK

## Abstract

**Background:**

Primaquine is currently the only medication used for radical cure of *Plasmodium vivax *infection. Unfortunately, its use is not without risk. Patients with glucose-6-phosphate dehydrogenase (G6PD) deficiency have an increased susceptibility to haemolysis when given primaquine. This potentially fatal clinical syndrome can be avoided if patients are tested for G6PD deficiency and adequately informed before being treated.

**Case presentation:**

A 35-year old male presented to our clinic on the Thai-Burmese border with a history and clinical examination consistent with intravascular haemolysis. The patient had been prescribed primaquine and chloroquine four days earlier for a *P. vivax *infection. The medication instructions had not been given in a language understood by the patient and he had not been tested for G6PD deficiency. The patient was not only G6PD deficient but misunderstood the instructions and took all his primaquine tablets together. With appropriate treatment the patient recovered and was discharged home a week later.

**Conclusions:**

Whilst primaquine remains the drug of choice to eradicate hypnozoites and control *P. vivax *transmission, the risks associated with its use must be minimized during its deployment. In areas where *P. vivax *exists, patients should be tested for G6PD deficiency and adequately informed before administration of primaquine.

## Background

*Plasmodium vivax *contributes to a significant proportion of malaria infections worldwide and it is the dominant species of malaria in many areas outside Africa[1]. Between 70 to 390 million infections are estimated to occur each year with about 80% of these infections occurring in South and South East Asia [2]. Unlike *Plasmodium falciparum*, the *P. vivax *parasite has a dormant liver stage [3]. Hypnozoites reside in the liver for prolonged periods of time before rupturing into the blood and causing a relapse of *P. vivax *infection. Once *P. vivax *has been eliminated from the bloodstream, the remaining hypnozoites must be eradicated from the liver to prevent relapse of infection. This is known as radical cure. Hypnozoites appear to be the dominant source of new parasitaemia and consequently further transmission of *P. vivax *malaria. Eradication of hypnozoites may, therefore, be an effective method for reducing the disease burden of *P. vivax*. Primaquine, an 8-aminoquinoline, is currently the only medication used for radical cure of *P. vivax*. It is recommended by the World Health Organization (WHO) to prevent relapse in patients infected with *P. vivax *malaria in areas of low transmission[4]. Despite this, due to many factors, only a minority of patients actually receive primaquine for *P. vivax *infection [5].

The use of primaquine is not without its risks. Patients with the inherited sex-linked deficiency of glucose-6-phosphate dehydrogenase (G6PD), have an increased susceptibility to acute intravascular haemolysis when treated with oxidant drugs such as primaquine [6]. Exposed patients commonly present with severe abdominal pain, nausea, vomiting and headache. High fevers with rigors can also be seen. The urine becomes almost black and output drops as renal failure ensues. This severe clinical syndrome of intravascular haemolysis, haemoglobinuria and acute renal failure is known as black water fever.

This potentially fatal clinical syndrome can be avoided if patients are tested for G6PD deficiency before the administration of primaquine. The most reliable way to detect G6PD deficiency is by DNA analysis, but a diagnosis of G6PD deficiency can also be made by a rapid fluorescent spot test [7]. The International Committee for Standardization in Haematology recommended this test as the most acceptable method for screening [8]. If the patient is deficient, the blood spot will fail to fluoresce under ultraviolet light (UV). The test is simple and inexpensive and, therefore, suitable for use in the field. It does however require a UV light source and, therefore, electricity, which can limit its use in resource poor settings. In response to this problem, other qualitative screening tests have recently been developed which do not require a UV light source, however they present other disadvantages in terms of cost, time and feasibility in the field [9], [10]. New rapid tests are being developed, which do not require special storage or performing conditions and couple malaria detection and G6PD activity assessment. These could represent a great tool for field analysis [11].

## Case presentation

A 35-year-old male was carried to SMRU malaria clinic on the Thai-Burmese border. He was confused, looked unwell, had rigors and was too weak to stand. His temperature was 37.4°C, he was tachypnoeic with a respiratory rate of 26/minute and had oxygen saturations of 83% on air. He had cold peripheries, a pulse rate of 93/min and a blood pressure of 80/65 mmHg.

His low oxygen saturations corrected with the administration of oxygen. Given the hypotension, tachycardia and pyrexia, both intravenous fluids and empirical ceftriaxone were begun.

Further assessment found pale conjunctiva and yellow sclera. His abdomen was diffusely tender but the liver and spleen were not palpable. His examination was otherwise unremarkable.

**Figure 1:**
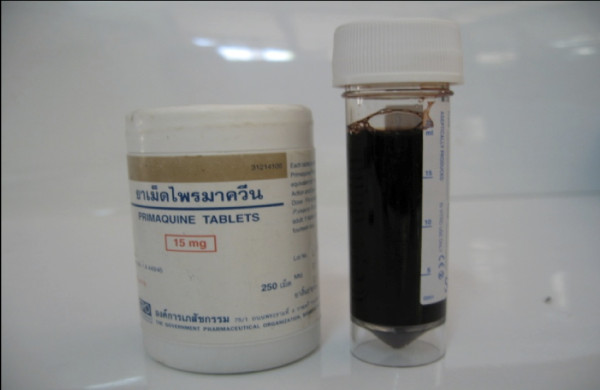
**A sample of the patient's urine at admission**. The black colour of the urine is due to the presence of haemoglobinuria.

His malaria smear was negative and he was normoglycaemic (193 mg/dl). However his capillary blood haematocrit (Hct) was only 18% and his urine sample was almost black (Figure 1). His urea and creatinine were elevated at 53.3 mg/dl and 2.1 mg/dl respectively. His full blood count showed white blood cell 21.6 x10^3 ^/μL, haemoglobin 6.9 g/dL and platelet count 136 × 10^3 ^/μL. It is possible that the leukocytosis could be a consequence of the haemolytic crisis itself [12]. Despite ceftriaxone therapy, the patient remained febrile with a leucocytosis at day three of admission. Given his clinical condition at this stage, gentamicin was added to his treatment and antibiotics were continued for a total of seven days. *Acinetobacter baumannii *was isolated from the admission blood culture: by disc testing using current CLSI criteria, the organism was sensitive to all drugs tested: amikacin, ceftazidime, ciprofloxacin, gentamicin, meropenem, and piperacillin-tazobactam. The clinical significance of this culture result is uncertain since the organism is usually responsible for infections in hospitalized patients [13].

Following initial treatment the patient became coherent enough to answer questions appropriately. He was a Burmese construction worker working on the Thai-Burmese border. Following a four-day history of fever, he attended a local clinic where a malaria smear confirmed he had malaria. He received two types of tablets, but was unsure of their names. The patient identified the tablets, using examples of anti-malarial medications, to be chloroquine and primaquine. As reportedly prescribed, the patient took three chloroquine tablets a day (10 mg/kg) and one primaquine tablet (15 mg base) a day for the first two days. On the third day he took one-and-a-half chloroquine tablets (5 mg/kg) and one more primaquine tablet (15 mg base). He did note a change in colour of his urine on the third day but was not alarmed by this. On day four he could not remember the instructions of what to do with the eleven remaining primaquine tablets. The handwritten instructions were in Thai, which he was unable to read. His colleague translated the label, telling him he must finish all the tablets. Subsequently, the patient took the last eleven primaquine tablets in one go (165 mg base). Later that day the patient began to vomit and a severe abdominal pain ensued associated with black urine.

**Figure 2:**
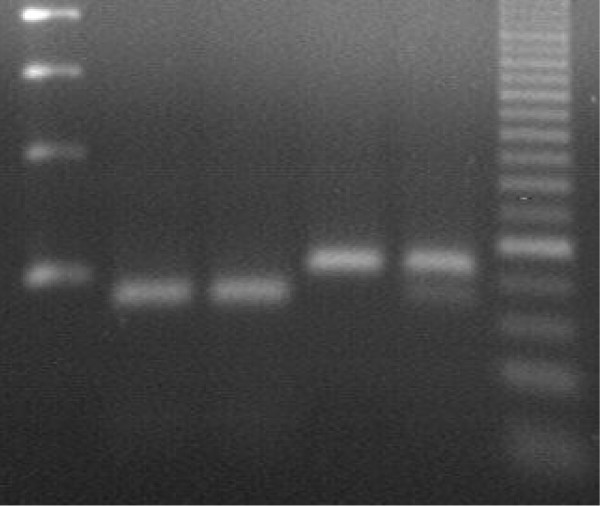
**Electrophoresis gel confirming the patient to be hemizygote for Mahidol variant of G6PD deficiency**. From the left side. Line 1: Molecular marker 100 bp; L2: Mahidol hemizygote; L3 Patient sample; L4:G6PD normal; L5: G6PD normal/Mahidol heterozygote; L6: molecular marker 25 bp.

Following this additional history, a second malaria smear was performed, which still showed no evidence of malaria. A rapid fluorescent spot test showed the patient to be G6PD deficient even though after a massive haemolysis, a normal phenotype would be expected as older red blood cells (RBCs) are destroyed and new ones, with normal phenotype, are produced. A correct protocol to avoid misinterpretation of the test after haemolysis, which has not been applied here, would have been to separate older RBCs and assess G6PD activity only on them. Genotyping through the PCR-restriction fragment length polymorphism (PCR-RFLP) method was later applied to detect known G6PD mutations [14]. This confirmed that the patient was hemizygote for the Mahidol variant (Figure 2).

After two 300 ml blood transfusions and ongoing intravenous fluids, the patients condition improved, his urine returned to normal and his Hct stabilized at 24%. At this level of anaemia, the local guidelines advise no further transfusion. The patient was discharged home on a treatment dose of ferrous sulphate (200 mg tds) and folic acid (5 mg od). A thorough explanation of the cause of his life-threatening illness was given to the patient in his own language and strict instructions, both verbal and written, explaining which medicines he should never take. Despite arranging follow-up to monitor the patient's renal function and anaemia, the patient did not return as he moved from the area.

## Conclusions

As G6PD deficiency confers an element of protection against infection with malaria, the two pathologies often exist side-by side in the same population [15,16]. In areas where *P. vivax *exists, the prevalence of the enzyme defect should be established, as it can be as high as 30% [17]. The rapid fluorescent spot test is a relatively inexpensive and simple test to run. Therefore testing for G6PD deficiency before administration of primaquine should be mandatory in areas where the deficiency is found to be common. Nonetheless the rapid test is qualitative and can be considered reliable only to detect complete deficient phenotypes. The interpretation of intermediate fluorescent test is more difficult and the decision about the treatment should follow the precautionary principle.

There are many different genotypes of G6PD deficiency. Each genotype is associated with a different level of enzyme deficiency and therefore susceptibility to haemolysis [18,19]. These different genotypes can only by identified by DNA analysis, which is not routinely available. What's more, within a given genotype there is a wide range of enzyme deficiency and therefore DNA analysis is still not entirely predictive of the haemolytic risk. To reduce the risk of haemolysis, the WHO currently makes the following recommendations: In patients with mild G6PD deficiency i.e. those patients with a history of tolerability of primaquine, the WHO suggests using an intermittent primaquine regimen of 0.75 mg base/kg once a week for eight weeks [4]. However, there is limited evidence to support this [20]. Primaquine is contraindicated in patients with severe glucose-6-phosphate dehydrogenase deficiency, therefore, in regions where prevalence of G6PD deficiency is relatively high, G6PD testing is required before administration of primaquine [4].

The exact prevalence of G6PD deficiency on the Thai-Burmese border is not yet fully characterized. However, the enzyme defect is known to be common in this area with G6PD Mahidol thought to be the most frequent mutation [19,21]. Research done by Buchachart *et al *suggests that administration of primaquine to patients with the Mahidol variant is relatively safe, but further research is needed [22]. Until these aspects of G6PD deficiency are better understood and the characterization of other deficient mutations in the local population is established (including their spatial distribution and the phenotypes associated with), in the absence of confirmed G6PD-normal status or evidence of previous primaquine tolerability, primaquine should not be administered.

The Thai Ministry of Public Health advocates using chloroquine and primaquine in the treatment of *P. vivax*, but gives no guidance on the use of G6PD testing [23]. Along the Thai-Burmese border, limited testing facilities for G6PD deficiency exist and primaquine is still prescribed in some clinics without confirming that the patient is G6PD normal. The consequence of such practice is demonstrated by this case and serves to remind that acute haemolysis is a real and potentially fatal consequence when primaquine is administered to patients with G6PD deficiency.

This case illustrates that low functional literacy can have serious consequences for individual health. Along the Thai-Burmese border, languages and dialects vary greatly according to the latitude. There is not one unique language spoken by all patients and medical staff in this area, making communication challenging. Furthermore there are high rates of illiteracy (<50%) in this area [24].

There is a strong association between patient literacy and adherence to malaria medication [25]. In order to ensure that malaria patients are receiving effective treatment, the role of the healthcare provider should go beyond the diagnosis and treatment of malaria and must include interventions that aid patient adherence and understanding [26]. The problem of illiteracy can be overcome by simple methods even when the majority of individuals are illiterate. It is essential that verbal instructions be given to the patients, as written instructions may be of limited use. Even with verbal instructions, patients with low literacy skills take words literally without interpreting them, so it is also imperative that a translator is used to ensure the full understanding of the patient [27]. A vital step in this process is to verify the patient's understanding of their illness and their medications by having them repeat back in their own language, what they are to do and why [28]. Clear visual instructions to take home are also important to support verbal communication. Studies have shown that pictorial aids improve understanding, and adherence, particularly when they are used together with oral instructions [29]. Visual aids are particularly useful for explaining when to take medications, and the importance of completing the therapy. At the very least, the patient should understand how to take his medication and when to seek further medical advice.

## Competing interests

The authors declare that they have no competing interests.

## Authors' contributions

KLB was responsible for the treatment of the patient, GB confirmed the G6PD deficiency genotype by PCR and provided photographic evidence of the diagnosis; KLB, GB and FN coordinated the manuscript. All authors read and approved the final manuscript.

## Consent

Written informed consent was obtained from the patient for publication of this case report and any accompanying images. A copy of the written consent is available for review by the Editor-in-Chief of this journal.
